# Report of the Obstetric Department of the Philadelphia Dispensary, for the Year 1843

**Published:** 1844-03-23

**Authors:** Joseph Warrington


*Report of the Obstetric Department of the Philadelphia Dispensary, for the year* 1843. By JOSEPH WARRINGTON, M. D.

Number of women delivered, at or near the full period of gestation, . . . . . 179

Number of children born, { males, 109 / females, 74 } Total, 183

A simple cell or vesicle is the very initiatory development, the *genesis* of every structure, in both the animal and vegetable kingdoms.—*Dr. Power.*

